# Stereotactic versus conventional radiotherapy for pain reduction and quality of life in spinal metastases: study protocol for a randomized controlled trial

**DOI:** 10.1186/s13063-016-1178-7

**Published:** 2016-02-02

**Authors:** Pètra Braam, Philippe Lambin, Johan Bussink

**Affiliations:** Department of Radiation Oncology, Radboud University Medical Center Nijmegen, Geert Grooteplein-Zuid 32, route 874, PB 9101, Nijmegen, GA 6525 The Netherlands; MAASTRO Clinic, Doctor Tanslaan 12, 6229 ET Maastricht, The Netherlands

**Keywords:** Stereotactic, Spinal metastases, SBRT, Pain, Quality of life, Palliative radiotherapy, IMRT

## Abstract

**Background:**

Painful spinal metastases have been treated with conventional radiotherapy for decades, but one-third of the patients have insufficient pain relief after treatment and one-fifth need retreatment. Stereotactic radiotherapy is a method to increase the dose in the spinal metastases with a potentially longer lasting palliative effect without increasing the side effects of the treatment and thereby is expected to improve the quality of life significantly.

**Methods/Design:**

This study is a multicenter prospective randomized clinical trial comparing conventional radiotherapy (1 x 8Gy) with stereotactic radiotherapy (1 x 20Gy) for pain reduction and quality of life in patients with painful spinal metastases. A total of 386 patients will be randomized between the two treatment groups. Besides pain measured by the Dutch Brief Pain Inventory, quality of life and cost-effectiveness also will be measured. The primary outcome is pain reduction at 6 weeks after treatment. Secondary outcomes will be the time to pain response, duration of pain relief, health-related quality of life and toxicity, as well as cost-effectiveness.

**Discussion:**

This study investigates whether stereotactic radiotherapy with dose escalation for symptomatic spinal metastases can lead to improved pain reduction as compared to conventional radiotherapy without an increase of treatment-related side effects. These results will contribute to the optimization and individualization of the treatment for the patient.

**Trial registration:**

ClinicalTrials.gov identifier NCT02407795 (March 31, 2015).

## Background

Many patients diagnosed with cancer have already metastasized disease or will metastasize in time. When there are no curative options, treatment will be focused on maintaining optimal quality of life. Up to 70 % of all bony metastases are located in the spinal column. Spinal vertebral metastases can give serious pain complaints, which are likely to progress in time and thereby decrease the quality of life [1]. Besides pain or risk of fracture, these metastases may also lead to spinal cord compression. Of all patients with spinal metastases, 8 to 20 % will develop symptomatic spinal cord compression, which, untreated, will lead to neurological complications such as the inability to walk or stand.

Conventional radiotherapy has been used for decades for palliation of pain caused by bone metastases. Patients are treated in a single or multiple fractions. Generally, a reduction in pain will occur in two-thirds of patients within 4 to 6 weeks after irradiation. Retreatment is necessary in up to 24 % of all patients [2]. Various studies, randomized or retrospective, have focused on dose and fractionation schemes in an effort to increase the efficacy of radiotherapy. The majority of these studies found no evidence that a single dose of 8Gy was inferior in pain relief compared to fractionated schedules for a higher irradiation dose, although the incidence of re-irradiation was higher using a single dose [2–7]. Because of these studies, in the Netherlands, a single dose of 8Gy is mainly used for painful metastatic lesions. In recent years, the results of systemic therapies improved significantly resulting in a better median survival of patients with metastatic disease. Therefore, patients nowadays that are irradiated to a single dose of 8Gy for spinal vertebral metastases are at higher risk of relapsing at the irradiated site. Re-irradiation can be offered to the patient; however, with conventional treatment, the radiation dose that can be given may be limited by the tolerance of the spinal cord.

Stereotactic body radiotherapy (SBRT) allows dose escalation to the spinal vertebra or paraspinal tumor with steep dose gradients that limit exposure to the spinal cord and other organs at risk. As the dose to the non-involved organs at risk is strongly reduced, fewer side effects will be expected compared to conventional radiotherapy. Due to the biologically higher dose that can be given to the target volume compared to conventional radiotherapy, better results are anticipated, especially when more radio-resistant tumors like renal carcinoma or melanoma are treated. Another question to be answered is whether we can identify a subgroup of patients that will benefit more from high dose stereotactic irradiation as compared to the conventionally used single fraction of 8Gy radiotherapy.

No data are available from randomized clinical trials comparing the standard 8Gy to SBRT high dose, nor are there dedicated phase I dose escalation studies. The total dose and the fractionation schemes have been mainly dependent on the physician [8–10]. The first patients treated by stereotactic radiotherapy for spinal metastases were reported in 1995. These patients were treated with multiple fractions. As time, and therefore experience, progressed, treatment techniques improved, and the number of indications for stereotactic radiotherapy increased. Currently, SBRT is a proven successful treatment modality for brain, liver, and lung tumors, as well as metastatic disease to these organs. Currently, one other active randomized trial in the United States is investigating single fraction conventional versus stereotactic radiotherapy for spinal metastases (RTOG 0631). In that trial, a single fraction of radiotherapy with conventional 8Gy is compared to stereotactic 16Gy, with primary endpoint pain relief at 3 months after treatment. Unfortunately, no time to response is measured, no consensus delineation rules have to be used, and no standardized quality of life questionnaires are used. Finally, if the trial is negative, it may be disputed whether 16Gy is sufficiently higher relative to 8Gy for obtaining a significant improvement in pain relief. Institutions already treating patients with stereotactic radiotherapy have reported doses of 20 to 24Gy as being a standard treatment, without serious toxicity [11]. However, when patients have been treated with a single fraction dose > 20Gy, an increased risk for vertebral compression fracture has been reported, especially in patients with baseline compression fractures, lytic tumors, or spinal misalignment [12, 13].

The aim of this study is to investigate whether stereotactic radiotherapy for symptomatic spinal metastases can lead to improved pain reduction as compared to conventional radiotherapy, without an increase of treatment related side effects and thereby result in a better quality of life. The primary objective is pain reduction, but we also focus on the duration of pain relief as an important secondary endpoint besides quality of life and cost-effectiveness. To answer these questions, an intensive scheme of time points for questionnaires and pain scores will be used. These results will contribute to the optimization and individualization of the treatment for the patient.

## Methods/Design

This is a multicenter, prospective randomized clinical trial to investigate pain reduction after radiotherapy. Two different radiation doses will be evaluated: 1 x 20Gy by SBRT and 1 x 8Gy conventional radiotherapy.

### Objectives/endpoints

The primary objective is to investigate whether stereotactic radiotherapy for symptomatic spinal metastases can lead to improved pain reduction as compared to conventional radiotherapy without an increase of treatment-related side effects. The primary endpoint will be pain reduction after treatment. Secondary endpoints are time to pain response, duration of pain relief, quality of life, and toxicity. In addition, cost-effectiveness will be studied.

### Recruitment and randomization

Patients must be able to undergo radiotherapy and fulfill the eligibility criteria. The diagnosis of a solid tumor must be pathologically confirmed to make the patient eligible for the study. The extent of the metastases in the spine should be visualized on an MRI, no longer than two weeks before randomization or no longer than 6 weeks before the start of treatment. To determine the clinically relevant category of stability, the Spinal Instability Neoplastic Score (SINS) will be obtained and documented prior to treatment. Earlier studies showed that the sensitivity of SINS for potentially unstable or unstable lesions was 95.7 %, with a specificity of 79.5 % [14, 15]. Written informed consent will be obtained from all participants before entering the study.

The study is designed to be carried out in multiple centers in the Netherlands. Patient randomization will only be allowed from authorized investigators, their authorized staff members, or data manager. A patient can be randomized only after verification of eligibility. In case a patient is eligible and a written informed consent is obtained, the randomization form must be faxed or digitally sent to the Data Center. Randomization will be performed using a password-protected database (OpenClinica). Patients will be stratified on primary tumor type (breast/prostate, non-small-cell lung/renal/colorectal/sarcoma/melanoma and others), Karnofsky performance status (60 to 70 versus 80 to 100) and number of spinal metastases (single lesion, 2 to 3 lesions, and disseminated osseous lesions).

### Inclusion and exclusion criteria

Inclusion criteria are as follows:

1. Histologically proven solid tumor with radiological diagnosis of spinal metastases
2. Pain score minimum 2 on an 11-point scale (0 = no pain to 10 = worst imaginable pain)
3. Maximum of two consecutive or noncontiguous spinal segments involved by tumor at current level of interest
4. Gross tumor optimally > 3 mm from the spinal cord on MRI
5. Karnofsky performance status ≥ 60
6. WHO 0-1-2
7. Life expectancy > 6 weeks
8. Age ≥ 18 years
9. Nonpregnant, nonlactating female patients. Sexually active patients of childbearing potential must implement effective contraceptive practices during the study
10. Written informed consent

Exclusion criteria are as follows:

1. History of previous radiotherapy to the spine at the current level of interest or overlapping location
2. Chemotherapy or targeted systemic therapy within 14 days of radiotherapy
3. Spinal instability or neurological deficit resulting from bony compression of neural structures
4. Pathologic fracture or impending fracture needing surgical fixation
5. Prior surgery to the spine at the current level of interest or overlapping location
6. Gross tumor < 3 mm from the spinal cord on MRI
7. More than 25 % spinal canal compromise
8. Patients with a pacemaker such that MRI cannot be performed or the treatment cannot be delivered safely
9. Patients not able to undergo MRI
10. Earlier nuclear medicine treatment for example strontium 89 treatment
11. Pregnancy
12. Altered mental status that would prohibit the understanding and giving of informed consent

### Assessment of the endpoints

Time to response is calculated from the date of randomization. Pain is self-reported by the patient and will be measured by an adjusted version for daily use of the Dutch Brief Pain Inventory (BPI). The BPI assesses pain on a 0–10 scale, where 0 is no pain and 10 is worst imaginable pain. It also has a questionnaire that consists of questions on severity of pain, impact of pain on daily function, location of pain, pain medications, and amount of pain relief in the past 24 hours or the past week. It is a short questionnaire that requires 5 minutes to complete. The BPI has been validated in more than 25 different languages. Response to treatment is calculated taking into account changes in the administration of opioids. Quality of life will be assessed using the EORTC QLQ-C15-PAL and the EORTC QLQ-BM22 [16–18]. These are well-validated, quality-of-life instruments specific to bone metastases. The EORTC QLQ-C15-PAL is a shortened version of the EORTC-C30, which is one of the most widely used questionnaires. The QLQ-C15-PAL consists of 15 items, and is the core questionnaire for palliative care. The QLQ-C15-PAL is supplemented by the disease specific module: the EORTC QLQ-BM22. This module is developed specifically for patients with bone metastases to evaluate the benefits and side effect of a treatment. It is intended to supplement the core questionnaire QLQ-C15-PAL and has completed all phases of testing as a reliable, validated, cross-cultural applicable module. The QLQ-BM22 contains 22 items divided into four scales assessing painful sites (five items), painful characteristics (three items), functional interference (eight items), and psychosocial aspects (six items). Both the QLQ-C15-PAL and the QLQ-BM22 questionnaires are patient rated because the gold standard for assessing symptom experience is self-reporting by the patient. The questionnaires will be available on paper or electronically, according to the wish of the patient. Toxicity is assessed using the CTC-AE 4.0. To measure the quality of the health status of the patients, the EuroQol-5D will be used [19]. In both randomization arms, the patients will be asked to fill out the following:

1. Days 0 to 14: their daily average, worst and present pain, intake of medication (dosage), and effect of medication (BPI)
2. Days 0, 7, and 14: effect of their pain on quality of life items (general activity, mood, walking ability, normal work, relations with other people, sleeping, enjoyment of life, and overall quality of life (EORTC-QOL)
3. Weeks 4 and 6 and months 2, 3, 4, 6, 9, 12, and thereafter, every 6 months with a maximum of 5 years or until death, both the BPI and QOL questionnaires. Concomitant use of analgesics should be noted.

Post-treatment diagnostics should be performed according to the institutions routine guidelines. Recurrence of disease should preferably be confirmed using MRI. Tumor growth less than 25 % is classified as stable disease. Data needed for re-irradiation and the occurrence of spinal cord compression and/or fractures, along with data on systemic treatment are collected at 3-month intervals at the institutes.

The pain score will be divided into four response categories for analyses. Complete response (CR) is defined as a pain score of 0 at the treated site, with no concomitant increase in analgesic intake. Partial response (PR) is defined as a pain reduction of 2 or more at the treated side on a scale of 0 to 10 without analgesic increase, or an analgesic reduction of 25 % or more from baseline without an increase in pain. Pain progression (PP) is defined as an increase in pain score of 2 or more above baseline at the treated site with stable analgesic intake or an increase of 25 % or more in analgesic intake compared with baseline with the pain score stable or 1 point above baseline. Stable pain (SP) is defined as any response that is not captured by the complete response, partial response, or pain progression definitions. Responders are defined as CR + PR; nonresponders are defined as PP + SP.

### Radiotherapy

For conventional radiotherapy, a single dose of 8Gy will be given using a linear accelerator with at least 6 MV photons. Preferably patients are treated using the ICRU guidelines, but there are no restrictions formulated with respect to the radiation technique. Patients should be treated according to the standard treatment protocol per institution.

For stereotactic treatment (1 x 20Gy), patients will be prepared with a dedicated CT simulator in treatment position with an immobilization device as used per institution. Standard position is supine, no prone position is allowed. Scans should be matched with the pretreatment (diagnostic) MRI images as they provide better delineation of the tumor and the spinal cord than the simulator CT scan. Target volumes are defined according to the ICRU Reports 50 and 62 and according to the International Spine Radiosurgery Consortium Consensus Guidelines for Target Volume Definition in Spinal Stereotactic Radiosurgery [20]. Dose-volume-histograms of the spinal cord or cauda equine, as well as from the organs at risk in the vicinity, will be obtained for all patients. For the spinal cord a maximum of 10Gy to 10 % of the spinal cord volume, which is defined as 6 mm above and below the target volume, is tolerated [21]. Circumferential radiation of the esophagus, trachea, great vessels, stomach, bowel, and rectum should be avoided. Patients should not receive chemotherapy or targeted systemic therapy within 14 days of radiotherapy. All palliative therapy and supportive care for disease-related symptoms will be offered to all patients on this trial. Details of these will be collected on case reports forms. Patients who do not achieve a response to radiotherapy, re-irradiation should only be considered 4 weeks after the initial treatment and only for the conventionally (8Gy) treated patients.

### Statistical analyses

The primary outcome of interest in this study is a reduction of pain of 2 points at 6 weeks since baseline. The literature shows that in patients with conventional radiotherapy the percentage of patients with pain reduction ranges from 66 % to 72 %. We assume that the percentage of patients with pain reduction is 70 % in the group with conventional radiotherapy compared to 85 % in the group with stereotactic radiotherapy. Therefore, 135 patients are needed in each group to obtain a power of 80 % (Fisher-exact test, two-sided, alpha = 0.05). We adjust for a drop-out rate of 30 % and will include a total of 386 patients. The primary analysis will be the comparison between the two treatment arms of the proportion of patients with pain reduction, defined as a decrease in pain score of at least 2 points from initial pain score, at 6 weeks. Time to pain progression, radiation-induced fracture and/or death will be calculated from date of randomization to the documentation. For the calculation of response, no fixed time interval from the date of randomization will be applied. Response of treatment will be calculated if at least two successive follow-up pain scores are available.

### Ethical issues, information, and safety

The protocol has been written and will be conducted according to the ICH Harmonized Tripartite Guideline for Good Clinical Practice. The study protocol was approved by the Central Dutch Medical Ethical Committee of the Radboud University Medical Center Nijmegen (2014). The regulations regarding medical confidentiality and data protection are fulfilled.

## Discussion

The aim of this trial is to test the hypothesis that stereotactic radiotherapy gives a better and longer lasting reduction of pain, better local control, and therefore a better quality of life without an increase of treatment-related side effects compared to conventional radiotherapy for symptomatic spinal metastases. Recent trials and reports using SBRT show excellent local control and a fast pain reduction in 1 to 2 weeks when treating painful spinal metastases [8, 10, 11, 22–26]. In recent years, systemic therapies have improved significantly, resulting in a better median survival of incurable patients, that is, with metastasized disease. Therefore, patients nowadays that are irradiated conventionally for spinal vertebral metastases are at a higher risk of local recurrence. The question is whether the conventional treatment is still appropriate for patients with potentially longer life expectancy, for example, patients with oligometastases or patients with tumors responsive to systemic treatments. Oligometastasis is defined as the state in which the patient shows distant relapse in only a limited number of regions. For these patients, a more radical approach could have a major impact on their quality of life and even life expectancy. In addition, stereotactic treatment shows a faster reduction of pain, occurring within 1 to 2 weeks, compared to pain reduction from conventional treatment, which usually occurs in 4 to 6 weeks. Therefore, patients with short life expectancies may also benefit from stereotactic treatment.

Quality of life is an important issue in cancer patients at all stages of disease. Quality of life is affected by many factors such as the disease itself, but also psychological and social factors. In patients with pain, the quality of life can be impaired due to the pain or the disease itself but also by the multiple treatments to which patients are exposed. The minimization of hospital visits is important. Furthermore, the analgesics can give side effects that may have a negative impact on the quality of life. Radiotherapy can reduce pain resulting from bone metastases and reduce analgesic use and it side effect, thereby improving the quality of life [1]. Knowledge of health-related quality of life and its implementation in the treatment options can help to guide patients in making decisions and personalize their treatment.

The question to be answered is whether we can identify a subgroup of patients that will benefit from a high dose stereotactic irradiation and consequently have a better quality of life as compared to the frequently used single fraction of low-dose conventional radiotherapy. This in order to optimize and to individualize the treatment options for the patient.

### Trial status

This trial began in May 2015 and is currently recruiting.

## Abbreviations

BPI: Brief Pain Inventory
CR: complete response
CT: computed tomography
CTC-AE: common terminology criteria for adverse events
ICRU: International Commission on Radiation Units and Measurements
MRI: magnetic resonance imaging
PP: pain progression
PR: partial response
QOL: quality of life
SBRT: stereotactic body radiotherapy
SINS: Spinal Instability Neoplastic Score
SP: stable pain
WHO: World Health Organization
